# Sesquiterpenes Are Agonists of the Pregnane X Receptor but Do Not Induce the Expression of Phase I Drug-Metabolizing Enzymes in the Human Liver

**DOI:** 10.3390/ijms20184562

**Published:** 2019-09-14

**Authors:** Michaela Šadibolová, Tomáš Zárybnický, Tomáš Smutný, Petr Pávek, Zdeněk Šubrt, Petra Matoušková, Lenka Skálová, Iva Boušová

**Affiliations:** 1 Department of Biochemical Sciences, Faculty of Pharmacy in Hradec Králové, Charles University, 500 05 Hradec Králové, Czech Republiczarybnto@faf.cuni.cz (T.Z.); matousp7@faf.cuni.cz (P.M.); skaloval@faf.cuni.cz (L.S.); 2 Department of Pharmacology and Toxicology, Faculty of Pharmacy in Hradec Králové, Charles University, 500 05 Hradec Králové, Czech Republic; smutt6aa@faf.cuni.cz (T.S.); pavek@faf.cuni.cz (P.P.); 3 Department of General Surgery, Third Faculty of Medicine and University Hospital Královské Vinohrady, Charles University, 100 34 Prague, Czech Republic; subrt@email.cz; 4 Department of Surgery, University Hospital Hradec Králové, 500 05 Hradec Králové, Czech Republic

**Keywords:** sesquiterpene, mRNA expression, protein expression, precision-cut liver slices, gene reporter assay, cytochrome P450 3A4, pregnane X receptor

## Abstract

Sesquiterpenes, the main components of plant essential oils, are bioactive compounds with numerous health-beneficial activities. Sesquiterpenes can interact with concomitantly administered drugs due to the modulation of drug-metabolizing enzymes (DMEs). The aim of this study was to evaluate the modulatory effects of six sesquiterpenes (farnesol, *cis*-nerolidol, *trans*-nerolidol, α-humulene, β-caryophyllene, and caryophyllene oxide) on the expression of four phase I DMEs (cytochrome P450 3A4 and 2C, carbonyl reductase 1, and aldo-keto reductase 1C) at both the mRNA and protein levels. For this purpose, human precision-cut liver slices (PCLS) prepared from 10 patients and transfected HepG2 cells were used. Western blotting, quantitative real-time PCR and reporter gene assays were employed in the analyses. In the reporter gene assays, all sesquiterpenes significantly induced cytochrome P450 3A4 expression via pregnane X receptor interaction. However in PCLS, their effects on the expression of all the tested DMEs at the mRNA and protein levels were mild or none. High inter-individual variabilities in the basal levels as well as in modulatory efficacy of the tested sesquiterpenes were observed, indicating a high probability of marked differences in the effects of these compounds among the general population. Nevertheless, it seems unlikely that the studied sesquiterpenes would remarkably influence the bioavailability and efficacy of concomitantly administered drugs.

## 1. Introduction

In view of promoting healthier living as well as addressing various health issues, the tendency of the population to seek out and use herbal remedies and nutraceuticals has significantly increased over the past years [1]. Moreover, the intake of natural remedies and herbal supplements concomitantly with prescription drugs has rapidly increased in terms of frequency. Although many of these herbal remedies have demonstrated a number of beneficial and health-promoting activities, information concerning possible herb–drug interactions is limited. Concurrent intake may indeed lead to undesirable herb–drug interactions at both the pharmacokinetic and pharmacodynamic level, which might result in adverse drug reactions with severe consequences [2,3]. Elderly people are particularly at risk, as more than other populations they suffer from various comorbidities and age-related pathophysiological changes as well as participate in polypharmaceutical treatment regimens that contribute to a reduction in drug clearance [4,5].

Recently, sesquiterpenes have drawn the attention of the research community owing to their considerable anti-inflammatory, antitumorigenic, antioxidant, and antiparasitic activities. Carrying these promising characteristics as the major components of plant essential oils, they are often present in various herbal medicines and nutraceuticals, especially widely used in the cosmetics and pharmaceutical industries [6]. Furthermore, as sesquiterpenes are inherent components of many spices, traditional delicacies, and beverages, they are often present in the human diet [7]. Their lipophilic character allows them to be easily absorbed following oral (or even topical) administration, with a single oral dose leading to measurable plasma concentrations [8,9,10].

We can infer from current evidence that sesquiterpenes are able to modulate the activity of some drug-metabolizing enzymes (DME) and could therefore interfere with the biotransformation of concurrently administered drugs despite the fact that information addressing this problem is scarce [6]. In a recent study, the cyclic sesquiterpenes α-humulene (HUM), β-caryophyllene (CAR), and caryophyllene oxide (CAO) were found to inhibit the activity of cytochrome P450 3A (CYP3A) in human and rat hepatic microsomes (with CAO being the strongest enzyme inhibitor), but did not affect the activities of other CYPs, carbonyl-reducing enzymes, or even conjugation enzymes [7]. In a similar manner, the linear sesquiterpenes farnesol (FAR), cis-nerolidol (cNER), and trans-nerolidol (tNER) inhibited the activities of some CYP isoforms, namely, CYP1A, CYP2B, and CYP3A, also in both human and rat hepatic microsomal fractions, but affected neither carbonyl-reducing enzymes nor conjugation enzymes [11]. Although sesquiterpenes inhibited CYPs activities in vitro, in vivo administration of CAO and tNER to mice increased CYPs expression and activity in the liver and small intestine [12].

**Figure 1:**
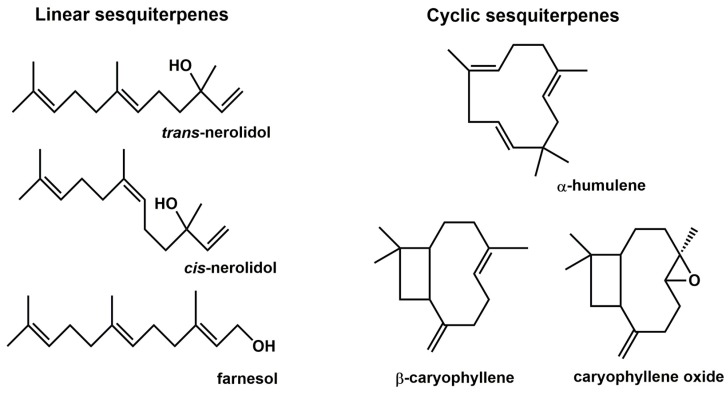
Chemical structures of the studied sesquiterpenes.

To address these seemingly contradictory findings, the present study was designed to develop more detailed information on the possible modulatory activity of six commonly used sesquiterpenes (HUM, CAR, CAO, FAR, cNER, and tNER; structures shown in Figure 1) on the expression of the main CYPs and carbonyl reducing enzymes. Human hepatic HepG2 cell line transfected with the human pregnane X receptor (PXR) and the aryl hydrocarbon receptor (AhR) along with human precision-cut liver slices (PCLS) were used for this purpose. Human PCLS represent a miniature in vitro tissue model which comprises all cell types of the tissue in their natural environment. PCLS have been successfully used in drug clearance, metabolism, and toxicity studies due to their relatively stable expression of DMEs and drug transporters [13,14]. Furthermore, their employment in enzyme induction studies has been substantiated [13,15]. Unlike cell culture models, PCLS preserve complex cell–cell and cell–matrix interactions, and are therefore more relevant in terms of reflecting the multicellular characteristics of the liver in vivo [14].

In our study, PCLS from 10 patients were incubated with sesquiterpenes, following which the changes in enzyme expression of CYP3A4, CYP2C, aldo-keto reductase 1C (AKR1C), and carbonyl reductase 1 (CBR1) were determined at the mRNA and protein levels. In addition, the sesquiterpenes were tested for their ability to interact with the receptors PXR and AhR which regulate the expression of two important CYP families, CYP3A and CYP1, respectively. This study contributes to the risk and safety assessment of the concomitant administration of the studied sesquiterpenes with prescription drugs.

## 2. Results and Discussion

The liver samples employed in this study were selected to be as healthy as possible, even with the varying clinical conditions of the patients. Human PCLS were prepared from liver samples obtained from ten patients undergoing partial hepatectomy for a malignant disease. Except for patient L6, plasma levels of total bilirubin (5.0–20.2 μkat/L), alanine aminotransferase (0.29–0.55 μkat/L), aspartate aminotransferase (0.32–0.47 μkat/L), and alkaline phosphatase (0.61–1.80 μkat/L), all of which provide information about liver functions, were within the physiological range in all patients. In patient L6, total bilirubin level was twice increased (48.9 μmol/L) along with a 3.6-times elevated level of conjugated bilirubin (12.3 μmol/L), which suggests the presence of intrahepatic biliary obstruction caused by ongoing malignancy [16]. The level of γ-glutamyltransferase (0.35–1.97 μkat/L) exceeded the physiological range 1.1–2.9-times in six out of the ten patients. The elevation in γ-glutamyltransferase levels is often seen in patients with biliary tract diseases including malignancies [17] as well as in patients with colorectal carcinoma with liver metastases [18]. Scoring of all liver samples for steatosis (score of 0–1) and fibrosis (score of zero) was performed by a pathologist. Based on the obtained scores, no or mild signs of liver disease were found in the patients’ biopsies.

### 2.1. The Effect of Sesquiterpenes on AhR and PXR Activation

Initially, all the sesquiterpenes were tested for the ability to activate the PXR and AhR nuclear receptors, which are known to be involved in the xenobiotic-induced increase of cytochrome P450 (CYP) 3A4 and CYP1A enzyme expression, respectively [19,20,21]. A luciferase reporter gene assay in HepG2 cells was employed for this purpose. The cells were treated with the sesquiterpenes in two concentrations (10 and 30 μM). Rifampicin (RIF, 10 μM) and methylcholanthrene (MC, 10 μM), well-known PXR and AhR ligands, respectively, were used as positive controls.

**Figure 2:**
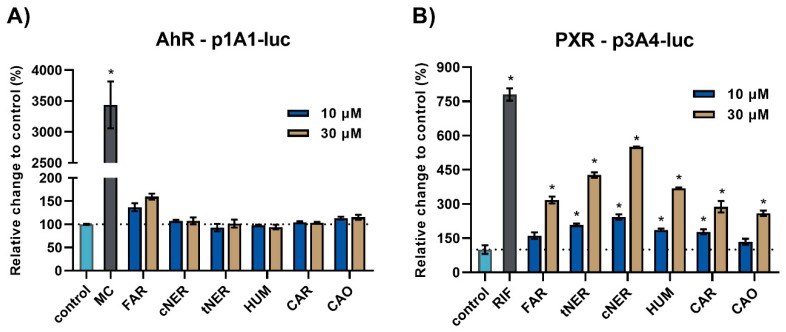
The effect of sesquiterpenes on the AhR and PXR receptors. HepG2 cells were transiently transfected with either p1A1-luc (**A**) or p3A4-luc in combination with expression vectors pSG5-PXR and pSG5-RXRα (**B**). The next day, the cells were treated with the tested compounds for 24 h. AhR and PXR as well as the well-known ligands methylcholanthrene (MC, 10 μM) and rifampicin (RIF, 10 μM) were used as positive controls. The samples were subsequently assayed by a Dual-Luciferase Reporter Assay System (Promega). The results are presented as the relative change to DMSO-treated controls defined as 100% (*n* = 3). * *p* < 0.05.

Neither of the tested compounds showed the potential to interact with the AhR nuclear receptor (Figure 2A), whereas marked interaction of MC was observed. On the other hand, all the sesquiterpenes were able to activate the PXR signaling pathway. The treatment with a higher concentration (30 μM) resulted in more pronounced PXR activation by all the studied compounds. However, tNER, cNER, HUM, and CAR managed to activate PXR even at lower concentrations (10 μM). The PXR activators tNER and cNER appeared to be the most potent at both the lower (2.4- and 2.1-fold, respectively) and higher (5.5- and 4.3-fold, respectively) concentrations (Figure 2B).

In our experiments, all the studied sesquiterpenes caused mild to intermediate activation of human PXR, reaching 2.6- to 5.5-fold at the 30 μM concentration. In another study, the sesquiterpenes zederone and germacrone caused the significant and dose-dependent activation of mouse PXR, while their effect on the activation of human PXR was weaker and comparable to our obtained results. In accordance with our findings, zederone and germacrone did not activate human AhR at 1–30 μM concentrations [22]. It has been reported that the antimalarial drug artemisinin and its derivatives were also able to moderately activate human PXR [23,24].

### 2.2. Basal mRNA and Protein Expression of CYP3A4, CYP2C, CBR1, and AKR1C3 in PCLS

Based on the results of the gene reporter assay, the effect of sesquiterpenes on the gene and protein expression of selected phase I DMEs have been studied in human PCLS. As none of the selected sesquiterpenes significantly activated the AhR-responsive luciferase construct, their effect on the expression of CYP1A1/2 has not been tested. Four major phase I DMEs, namely CYP3A4, CYP2C, CBR1, and AKR1C3, were selected. Both CYP3A4 and CYP2C, the most abundant CYPS in the human liver and the main DME involved in oxidative biotransformation of drugs, are downstream targets of PXR/CAR nuclear receptors [25]. The transcription regulation of CBR1 and AKR1C, the main DME for drugs bearing the carbonyl group, proceeds mainly by the nuclear factor erythroid 2-related factor 2 (Nrf2) system via the antioxidant-response element (ARE), which is present in their gene promotor [26,27]. As several sesquiterpenes and sesquiterpene lactones have been reported to activate the Nrf2-ARE-dependent detoxification pathway [28,29], CBR1 and AKR1C expression was tested in the present study.

**Figure 3:**
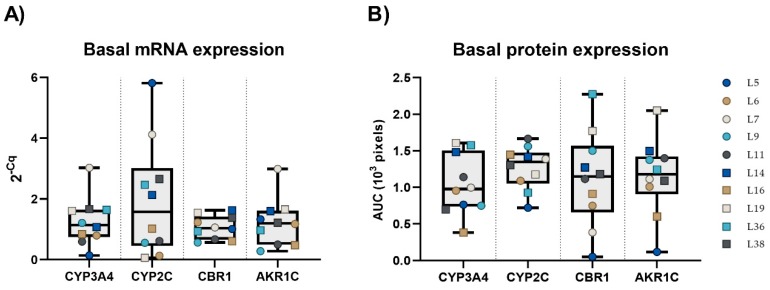
Inter-individual variability in the basal expression of selected mRNAs (**A**) and proteins (**B**) in PCLS from ten patients. The horizontal line represents the median, and whiskers represent the maximum and minimum values.

In the control PCLS, basal expressions of four selected DMEs at the mRNA and protein level were measured. Concerning mRNA expression, CYP2C was the DME with the highest variability, while CBR1 was the most stably expressed gene (Figure 3A). The mRNA levels of CYP2C and CBR1 among samples with the lowest and the highest expression differed 92.2-times and 2.9-times, respectively. With regards to protein expression, the situation was reversed and CBR1 exerted the highest variability among the studied enzymes, while CYP2C was stably expressed in all liver samples (Figure 3B).

In our results, marked inter-individual differences in the basal expression of all the selected DMEs among the individual liver samples were observed. However, a good correlation between mRNA levels of CYP3A4 and AKR1C (*r* = 0.688, *p* = 0.0278), the protein levels of CYP3A4 and AKR1C3 (*r* = 0.699, *p* = 0.0244), and the protein levels of CBR1 and AKR1C3 (*r* = 0.691, *p* = 0.0248) were all found in human PCLS (untreated controls). A meta-analysis of 50 studies dealing with the abundance of human hepatic cytochrome P450 enzymes in Caucasian adult livers showed a strong positive correlation between the expression levels of CYP3A4 and CYP2C8/9 [30]. As was reported previously, the PCLS represent individuals exhibiting large variations in basal mRNA levels as well as in responsiveness to potential inducers [15,31,32].

### 2.3. The Effect of Sesquiterpenes on the mRNA Expression of the Studied Enzymes

As sesquiterpenes are important components of popular nutraceuticals and dietary supplements, their ability to modulate the activity and/or expression of DMEs and drug transporters becomes an important question. Recently, the inhibitory effect of linear (cNER, tNER, and FAR) and cyclic (HUM, CAR, and CAO) sesquiterpenes on the activity of the CYP3A subfamily in human and rat hepatic subcellular fractions was observed, while the activities of carbonyl-reducing and conjugating enzymes were not significantly influenced [7,11]. In human liver microsomes, other sesquiterpenes, zederone and germacrone, moderately inhibited CYP2B6 and CYP3A4 activities, with IC_50_ values below 10 μM [22]. The sesquiterpene lactone alantolactone acted as non-competitive inhibitor of CYP3A4 in human liver microsomes, with an IC_50_ equal to 3.6 μM [33]. On the other hand, a marked increase in CYP2B and CYP3A activity as well as in mRNA levels was observed after 24 h in the liver and small intestine of mice orally treated with tNER and CAO (50 mg/kg) [12].

In the present study, the effect of FAR, tNER, cNER, HUM, CAR, and CAO on the mRNA expression of CYP3A4, CYP2C, CBR1, and AKR1C was studied in human PCLS prepared from 10 liver samples. As the number of PCLS prepared from one tissue sample was insufficient for testing all six sesquiterpenes, five samples were used for the cyclic sesquiterpenes and five samples for the linear ones. RIF was used in all PCLS as a positive control. The PCLS were incubated in the presence of individual sesquiterpenes, DMSO (control), and RIF (positive control) for 24 h. PCR primers for CYP2C and AKR1C were designed to amplify all four human CYP2C isoforms (namely 2C8, 2C9, 2C18, and 2C19) and all four AKR1C isoforms (namely AKR1C1–4), respectively.

**Figure 4:**
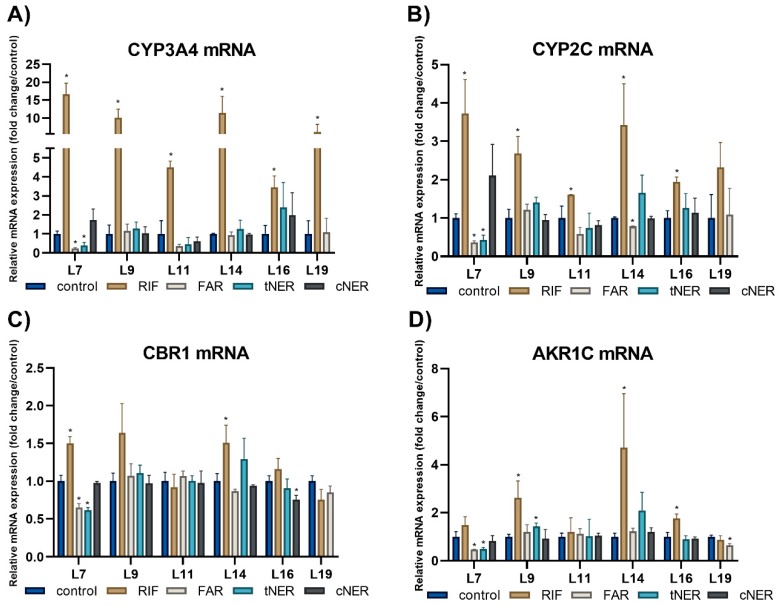
Inter-individual differences in the effect of linear sesquiterpenes (10 μM) and RIF (10 μM) on the normalized mRNA expression of CYP3A4 (**A**), CYP2C (**B**), CBR1 (**C**) and AKR1C (**D**) in human PCLS from five patients after 24 h (*n* = 3). The normalized expression level was calculated using the 2^−ΔΔCt^ method with the geometric mean of GAPDH and SDHA as a reference gene. Results are presented as the mean ± SD (*n* = 3). Statistical analyses were performed using one-way ANOVA with Dunnett’s test: *p* < 0.05 (*).

The linear sesquiterpenes FAR, cNER, and tNER showed some effects on the mRNA expression of DMEs. Results are presented in Figure 4. In patient L7, FAR and tNER caused a significant decrease in the mRNA level of all four studied enzymes. This inhibitory effect was most pronounced in the case of CYP3A4, in which FAR and tNER reduced the level of mRNA by 76.3% and 60.8%, respectively. In this patient, basal expression of CYP3A4, CYP2C, and AKR1C ranked among the highest expression levels. In patient L9, tNER induced AKR1C expression 1.4-times. Taken together, FAR significantly influenced the expression of CYP3A4 and CBR1 in one patient and the expression of CYP2C and AKR1C in two patients; cNER reduced only CBR1 expression in one patient; while tNER inhibited the mRNA level of CYP3A4, CYP2C, CBR1, and AKR1C in one patient and induced AKR1C expression in another. In contrast to the linear sesquiterpenes, the cyclic sesquiterpenes HUM, CAR, and CAO showed no significant effect on the mRNA expression of all the studied DMEs (Figure S1). The applicability of the chosen model system was proved by 10 μM RIF (positive control, a prototypical ligand of the human PXR), which caused significant induction on CYP3A4 expression in PCLS from all patients.

In human PCLS, mRNA expression of the studied DMEs was only mildly influenced by the tested sesquiterpenes. Their effect is noticeably lower than might be expected based on the results of the gene reporter assay, in which 10 μM cNER induced the mRNA level of CYP3A4 2.4-times. This discrepancy can be explained by the different nature of model systems, i.e., HepG2 cells and PCLS. In human PCLS, the AhR-, PXR- and CAR-mediated induction of major CYP mRNAs can be detected, although the extent of the induction is often lower than in the primary hepatocytes [15,31,32]. Moreover, inter-individual variability in responses is often seen when using PCLS as a model system [34]. However, PCLS represent a miniature model of liver tissue with preserved cell–cell and cell–matrix interactions and all cell types are present, therefore, PCLS are more relevant to a physiological state than cells in a cell culture. On the other hand, HepG2 cells possess very low basal CYPs enzymatic activity. Therefore, this discrepancy may be explained by the fast degradation of sesquiterpenes in liver slices, but not in the HepG2 cells.

Various factors can contribute to inter-individual variability in the response to administered drugs/compounds. One of them is a level of constitutive expression of individual DMEs and nuclear receptors, which is affected by sex, genetic polymorphism, food, environmental factors, medication, pathological conditions etc. In addition, the induction effect could be influenced by the level of sesquiterpene, which depends on the rate of its metabolism. For example, three metabolites of farnesol (i.e., hydroxyfarnesol, farnesyl glucuronide and hydroxyfarnesyl glucuronide) have been identified in human liver microsomes [35]. If such metabolites of sesquiterpenes are less active in DME induction than parent compounds, higher effect of sesquiterpenes in some PCLS could be attributed to lower activity of CYP and/or UGT in those individuals.

In some PCLS, inhibitory effect of FAR and tNER was observed. One of the mechanisms, which could explain sesquiterpenes-mediated inhibition of DMEs’ gene expression, could be based on their possible involvement in the epigenetic regulation of those genes. As was reported earlier, sesquiterpene lactone parthenolide influenced the level of DNA methylation by decreasing expression and activity of human DNA methyltransferase 1 in several human cell lines and also affected chromatin remodeling by downregulation of histone deacetylase 1 level via a proteasome-dependent degradation [36,37]. In addition, expression of DMEs can be regulated by miRNAs either directly or indirectly by targeting DME regulators (e.g., nuclear receptors) [38]. Regulation of miRNA expression by several sesquiterpenes have been described [39,40], however, no report describing sesquiterpene-miRNA-PXR interaction was found.

### 2.4. The Effect of Sesquiterpenes on the Protein Expression of Studied Enzymes

Subsequently, the influence of the studied sesquiterpenes on the protein expression of DMEs was studied in human PCLS. Homogenates from individual PCLS, which were treated in the same way as was the case in the mRNA expression study, were prepared and pooled. Calnexin, protein present in the endoplasmic reticulum was used as a loading control. The primary antibodies against CYP3A4, i.e., CBR1, AKR1C3, and CYP2C8 + 2C9 + 2C19 + 2C12, were used to detect protein expression of the corresponding DMEs.

**Figure 5:**
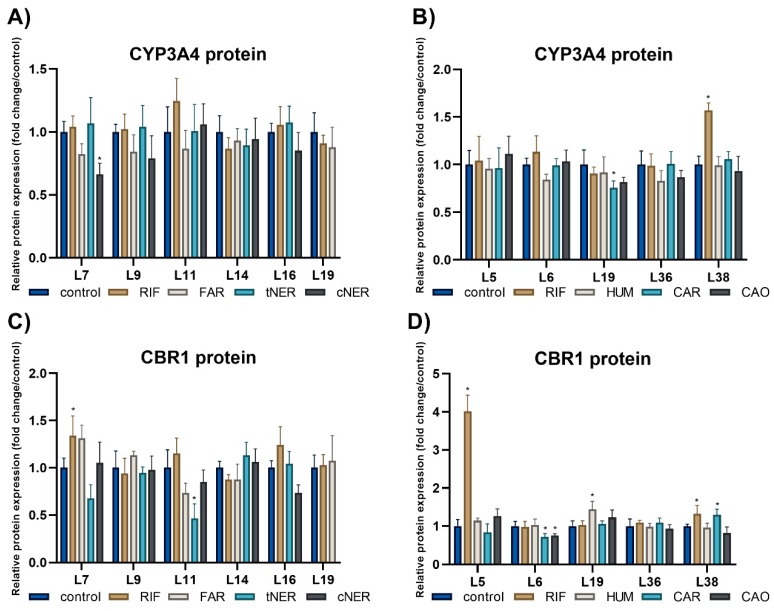
Inter-individual differences in the effect of sesquiterpenes (10 μM) and RIF (10 μM) on the normalized protein expression of CYP3A4 (**A**,**B**) and CBR1 (**C**,**D**) in human PCLS from ten patients after 24 h (*n* = 3). The protein expression was calculated using calnexin as a loading control. Results are presented as the mean ± SD (*n* = 4), with controls set to 100%. Statistical analyses were performed using one-way ANOVA with Dunnett’s test: *p* < 0.05 (*).

The studied sesquiterpenes possessed mild or no inhibitory influence on the protein expression of DMEs in individual patients. The highest inhibition was observed in the case of sample L11, in which tNER reduced the protein expression of CBR1 by 53.5%. On the other hand, this sesquiterpene increased the protein expression of CYP2C and AKR1C3 1.36-times and 1.45-times, respectively, in sample L7 (Figure S2). The effects of individual sesquiterpenes differed among individual liver samples and DMEs, e.g., CAR elevated CBR1 expression in L38, while it decreased CBR1 level in sample L6. Results are presented in Figure 5.

The observed effect of the studied sesquiterpenes on the protein expression of the four DMEs was only weak, but certain inter-individual variability was found. However, the changes in the protein expression of DMEs are probably not biologically relevant. Knowledge regarding the sesquiterpenes‘ effects on the DMEs protein expression is scarce, as the effects have been studied only sporadically. For example, sesquiterpene lactone deoxyelephantopin (10 μM) showed no effect on the CYP3A4 protein expression, while the enzymatic activity of CYP3A4 was reduced by 45% in HepG2 cells [41].

## 3. Materials and Methods

### 3.1. Chemicals and Reagents

Sesquiterpenes α-humulene, β-caryophyllene (CAR), caryophyllene oxide (CAO), farnesol (FAR), *cis*-nerolidol (cNER) and *trans*-nerolidol (tNER), rifampicin (RIF), methylcholanthrene (MC) and fetal bovine serum (FBS) were purchased from Sigma Aldrich (Prague, Czech Republic). All other chemicals were of analytical grade or higher. Stock solutions of sesquiterpenes (10 mM) were prepared in dimethyl sulfoxide (DMSO) and stored at 4 °C in the dark.

### 3.2. Cell Culture

Human hepatoblastoma-derived (HepG2) cells were purchased from the European Collection of Authenticated Cell Cultures (ECACC, Salisbury, UK) and maintained in antibiotic-free Dulbecco’s modified Eagle’s medium (Thermo Fisher Scientific, Waltham, MA, USA) supplemented with 10% FBS at 37 °C in a humidified incubator under 5% CO_2_.

### 3.3. Plasmids

The expression plasmid encoding human PXR receptor (pSG5-PXR) was a generous gift from Dr. S. Kliewer (University of Texas, Dallas, TX, USA) and the pSG5-RXRα construct was kindly provided by Dr. C. Carlberg (University of Kuopio, Kuopio, Finland), while pRL-TK was obtained from Promega (Madison, WI, USA). The p3A4-luc reporter vector carries a distal XREM (–7836/–7208) and a basal promoter sequence (prPXRE, –362/+53) of the *CYP3A4* gene 5′-flanking region inserted to pGL3-Basic reporter vector [42]. The p1A1-luc plasmid bears the promoter region (–1566 to +73) of human *CYP1A1* gene [43].

### 3.4. Luciferase Reporter Gene Assays

The HepG2 cells were seeded into 48-well plates (30 000 cells/well) overnight and transfected either with gene reporter vector p1A1-luc (150 ng/well) or p3A4-luc (150 ng/well) in combination with expression vectors pSG5-PXR (100 ng/well) and pSG5-RXRα (50 ng/well), and co-transfected with pRL-TK (30 ng/well) for transfection normalization. The transfection was performed by Lipofectamine 3000 Reagent (Thermo Fisher Scientific, Waltham, MA, USA) following the manufacturer’s recommendations. After 24 h, the HepG2 cells were treated with the tested compounds at the indicated concentrations for an additional 24 h. The compounds were diluted in Opti-MEM I Reduced Serum Medium (Thermo Fisher Scientific, Waltham, MA, USA) supplemented with 5% FBS. Final concentration of the vehicle (DMSO) in media did not exceed 0.1% in all the experiments. After treatment, the cells were lysed and measured for both firefly and *Renilla* luciferase activities using a Dual-Luciferase Reporter Assay System (Promega, Madison, WI, USA).

### 3.5. Ethics Committee Statement

All the experimental procedures were approved by the Ethics Committee of the University Hospital Hradec Králové, Czech Republic (Permission No. 201703 S14P, 2 March 2017). An informed consent for tissue procurement for research purposes was obtained from all subjects.

### 3.6. Human Liver Tissue

Human liver tissue was provided by the University Hospital Hradec Králové as healthy surplus tissue from 10 patients (5 males and 5 females, 45–81 years old) undergoing partial hepatectomy due to the presence of a tumor. Table 1 summarizes a brief medical history of the liver tissue donors. The resected liver tissue was placed directly into a chilled vessel with Euro–Collins solution and transported to the laboratory for immediate handling. The liver tissue was regarded as healthy based on the results of biochemical tests and a histopathological examination. Routine biochemical tests (i.e., plasma levels of bilirubin, alanine aminotransferase, aspartate aminotransferase, γ-glutamyltransferase and alkaline phosphatase) were performed before the surgery. Histopathological examination of the liver tissue for signs of fibrosis and/or steatosis was performed by a pathologist.

### 3.7. Preparation of Precision-Cut Liver Slices and Experimental Treatment

The liver slices were prepared as described previously [44]. Briefly, small cylindrical cores were cut out of the liver tissue and sliced using the Krumdieck tissue slicer MD4000 (Alabama Research and Development, Munford, AL, USA) filled with an ice-cold Krebs–Henseleit buffer saturated with carbogen and containing 25 mM d-glucose, 25 mM NaHCO_3_, and 10 mM HEPES (Carl Roth, Karlsruhe, Germany). The liver slices (8 mm in diameter, 150–170 μm in thickness) were preincubated individually in 1 mL of Williams’ Medium E (with L-glutamine, Invitrogen, Paisley, UK) supplemented with 25 mM d-glucose and 50 μg/mL gentamycin in 12-well plates under continuous supply of 85% O_2_ and 5% CO_2_ with continuous shaking (90 times/min) at 37 °C for 60 min. Afterwards, the liver slices were transferred to new 12-well plates and incubated individually in 1.3 mL of fresh Williams‘ Medium E supplemented by either the tested compounds or DMSO (control) for 24 h. The final DMSO concentration did not exceed 0.2%. Due to the limited number of liver slices that could be prepared from one tissue sample, three linear (FAR, tNER, and cNER) and three cyclic (HUM, CAR, CAO) sesquiterpenes were studied separately. All the experiments were performed in triplicates using the liver tissue from five different patients.

### 3.8. RNA Isolation, cDNA Synthesis and Quantitative Real-Time PCR (RT-qPCR)

The liver slices were collected after 24 h of incubation. All treatments were performed in triplicates and every slice was placed separately into 500 μL of TriReagent and stored at −80 °C until use. Total RNA from every slice was isolated using TriReagent according to the manufacturer’s instructions (Biotech, Praha, Czech Republic). The homogenization of each sample was performed using a single steel bead in a 2 mL Eppendorf tube using a microhomogenizer. The purified RNA was dissolved in 40 μL of diethyl pyrocarbonate (DEPC)-treated water (0.01% DEPC in HPLC water, autoclaved) and stored at −80 °C. The measurement of the absorbance at 260 and 280 nm using the NanoDrop ND-1000 UV–vis Spectrophotometer (Thermo Fisher Scientific, Pardubice, Czech Republic) was used to determine RNA yields and purity. Subsequently, RNA (4 μg) was treated with 2 U of DNase I (New England Biolabs, Ipswich, MA, USA) in a final volume of 30 μL for 20 min at 37 °C, 1.5 μL of 0.1 M EDTA was added and the DNAse was inactivated by heat (10 min at 75 °C). The solution was diluted to a concentration of 0.2 μg/μL by adding 8.5 μL of DEPC water. The DNAse I treated RNA was stored at −80 °C until further analyses. The first strand cDNA was synthesized from 1 μg of total RNA and 1 μL of 50 μM random hexamers (Generi Biotech, Hradec Kralove, Czech Republic) using ProtoScript II reverse transcriptase (New England Biolabs, Ipswich, MA, USA). After initial heat denaturation of total RNA (65 °C for 5 min), 4 μL 5× ProtoScript II RT Reaction Buffer, 2 μL 10× DTT, 2 μL dNTP Mix 5 mM, 3.5 μL H_2_O and 0.5 μL ProtoScript II 200 U/μL were added and mixed by pipetting. The reactions (20 μL) were incubated for 10 min at 25 °C, for 50 min at 42 °C and for 5 min at 80 °C. The obtained cDNAs were diluted 1:6 by DEPC water. The qPCR analyses were carried out using QuantStudio 6 Flex (Applied Biosystems, Foster City, CA, USA) with SYBR green I (Xceed qPCR SG Mix, Institute of Applied Biotechnologies, Prague, Czech Republic) detection according to the manufacturer’s protocol. The samples contained both forward and reverse primers (both 250 nM) and 5 μL of diluted cDNA. Primer sequences are listed in Table 2. The PCR reactions started with a denaturation step (10 min, 95 °C) followed by 40 cycles of amplification which consisted of denaturation (10 s, 95 °C) and annealing and extension (40 s, 60 °C). Fluorescence data were recorded at the end of each amplification step. Relative expression levels of the target genes were calculated as fold changes in triplicates for each group using the 2^−ΔΔCt^ method. [45]. The normalized expression level was expressed using a geometric mean of reference genes (glyceraldehyde 3-phosphate dehydrogenase, GAPDH; subunit A of succinate dehydrogenase complex, SDHA).

### 3.9. Western Blotting

The liver slices were collected after 24 h of incubation. All treatments were performed in triplicates, with every slice was placed separately into 500 μL of lysis buffer and stored at −80 °C until use. Equal volumes of once homogenized and centrifuged triplicate samples were pooled together. Protein concentration was measured using the BCA protein assay (Sigma Aldrich, Prague, Czech Republic) according to the manufacturer‘s instructions. The proteins (25 μg) were loaded onto a 10% sodium dodecyl sulfate (SDS; *w*/*v*)–polyacrylamide gel (with 4% stacking gel) and separated by SDS-PAGE electrophoresis. The proteins were transferred onto a nitrocellulose membrane using the Trans-Blot Turbo Transfer System (Bio-Rad, Hercules, CA, USA). The membrane blocking was performed in a 5% non-fat dry milk/TRIS-buffered saline-Tween-20 (TBS-T) solution at room temperature for 2 h. Incubation with primary antibodies was accomplished overnight at 4 °C. Following primary antibodies were employed: Anti-Calnexin (ab75801, 1:2000), anti-AKR1C3 (ab27491, 0.1 μg/mL), anti-CBR1 (ab4148, 1:5000), anti-CYP2C (ab22596, 1:1000) (Abcam, Cambridge, UK), and anti-CYP3A4 (NB600-1396, 1:10,000) (Novus Biologicals, Cambridge, UK). Calnexin, a housekeeping protein, was used as a loading control. Afterwards, the membrane was washed with 0.3% TBS-T solution for 6 × 5 min, incubated with respective secondary antibodies conjugated with horseradish peroxidase (bovine anti-rabbit (sc2370) and bovine anti-goat (sc2350), 1:10,000) (Santa Cruz Biotechnology, Santa Cruz, CA, USA) at room temperature for 1 h, and rinsed with TBS-T solution for 6 × 5 min. Visualization of protein bands was carried out using the chemiluminescence kit (GE Healthcare, Buckinghamshire, UK) and Carestream BioMax light film (Sigma Aldrich, Prague, Czech Republic). The relative protein signal intensities were determined densitometrically using ImageJ software (National Institutes of Health, Bethesda, MD, USA).

### 3.10. Statistical Analysis

All calculations were performed in Microsoft Excel and GraphPad Prism 8 (GraphPad Software, San Diego, CA, USA). The results of the luciferase reporter gene assays are presented as the relative change in *Renilla*-normalized firefly luciferase activities compared to the vehicle-treated control activities set as 100%. The presented results are based on at least three independent experiments (*n* = 3). A *p*-value of < 0.05 was considered to be statistically significant.

In the RT-qPCR analysis, three liver samples (a triplicate) were measured individually, whereas in the Western blot analysis, a pooled sample was prepared from a triplicate and the experiment was repeated four times. The results are expressed as the mean ± SD. One-way ANOVA followed by Dunnett‘s post hoc test was used for the statistical evaluation of differences between the treated samples and control. Differences of *p* < 0.05 were considered as statistically significant.

## 4. Conclusions

Despite the fact that the studied sesquiterpenes acted as agonists of the PXR receptor, they possessed only a weak or no influence on the expression of CYP3A4, CYP2C, CBR1, and AKR1C at both the mRNA and protein levels. Moreover, high inter-individual variability both in the basal levels of the tested enzymes and in the modulatory effect of the sesquiterpenes in individual PCLS were observed. It seems improbable that the studied sesquiterpenes could significantly influence the bioavailability and efficacy of concomitantly administered drugs. Therefore, serious herb–drug interactions with the studied sesquiterpenes are not expected.

## Figures and Tables

**Table 1:** Brief medical history of liver tissue donors.

Liver Sample	Sex (Age)	Reason of Surgery	Comorbidities	Long-Term Pharmacotherapy
**L5**	male (63)	Colorectal carcinoma	HTN, hyperuricemia, type 2 DM	Ramipril, atorvastatin, metformin, allopurinol
**L6**	male (69)	Colorectal carcinoma	HTN	Hydrochlorothiazide
**L7**	male (69)	Colorectal carcinoma	HTN, s/*p* CVA	Acetylsalicylic acid, nitrendipine
**L9**	male (81)	Colorectal carcinoma	HTN, dyslipidemia	Betaxolol
**L11**	female (57)	Colorectal carcinoma	none	none
**L14**	female (45)	Benign focal nodular hyperplasia	none	none
**L16**	female (59)	Colorectal carcinoma	HLD, ovarian cancer	none
**L19**	female (65)	Colorectal carcinoma	HTN, HLD, impaired glucose tolerance	Amlodipine
**L36**	female (78)	Cholangiocellular carcinoma	HTN, HLD, coronary artery disease, atrial fibrillation	Bisoprolol, furosemide, ramipril, simvastatin, enoxaparin, zolpidem
**L38**	male (59)	Cholangiocellular carcinoma	none	none

HTN, arterial hypertension; DM, diabetes mellitus; *s*/*p* CVA, status post cerebrovascular accident; HLD, hyperlipidemia.

**Table 2:** List of primers used for RT-qPCR analysis of the selected genes.

Gene	Forward Primer	Reverse Primer
CYP3A4	CCCCTGAAATTAAGCTTAGGAGG	CTGGTGTTCTCAGGCACAGA
CYP2C	TTTGGGATGGGGAAGAGGAG	GGAGCACAGCCCAGGAT
CBR1	TTGGTACCCGAGATGTGTGC	CTTGGGGTTTTATTAGAGGGAG
AKR1C	ATGAGGAGCAGGTTGGACTG	GCTTTGAAGTGTAGAATATGTCTTCT

## Supplementary Materials

Supplementary materials can be found at https://www.mdpi.com/1422-0067/20/18/4562/s1.

## Abbreviations

AKR1C	aldo-keto reductase 1C
CAO	caryophyllene oxide
CAR	β-caryophyllene
CBR1	carbonyl reductase 1
cNER	*cis*-nerolidol
CYP	cytochrome P450
DEPC	diethyl pyrocarbonate
DM	diabetes mellitus
DME	drug-metabolizing enzyme
DMSO	dimethyl sulfoxide
FAR	farnesol
FBS	fetal bovine serum
GADPH	glyceraldehyde 3-phosphate dehydrogenase
HLD	hyperlipidemia
HUM	α-humulene
HTN	hypertension
MC	methylcholanthrene
RIF	rifampicin
RT-qPCR	reverse transcription-quantitative polymerase chain reaction
SDHA	succinate dehydrogenase complex, subunit A
s/*p* CVA	*status post* cerebrovascular accident
SDS-PAGE	sodium dodecyl sulfate–polyacrylamide gel electrophoresis
TBST-T	TRIS-buffered saline-Tween-20
tNER	*trans*-nerolidol
