# Supplementary material for: Sesquiterpenes Are Agonists of the Pregnane X Receptor but Do Not Induce the Expression of Phase I Drug-Metabolizing Enzymes in the Human Liver
Source: Int J Mol Sci. 2019 Sep 14;20(18):4562. doi: 10.3390/ijms20184562 (PMC6769599; doi:10.3390/ijms20184562)
Supplement: Supplementary file 1 [file ijms-20-04562-s001.pdf]

Supplementary

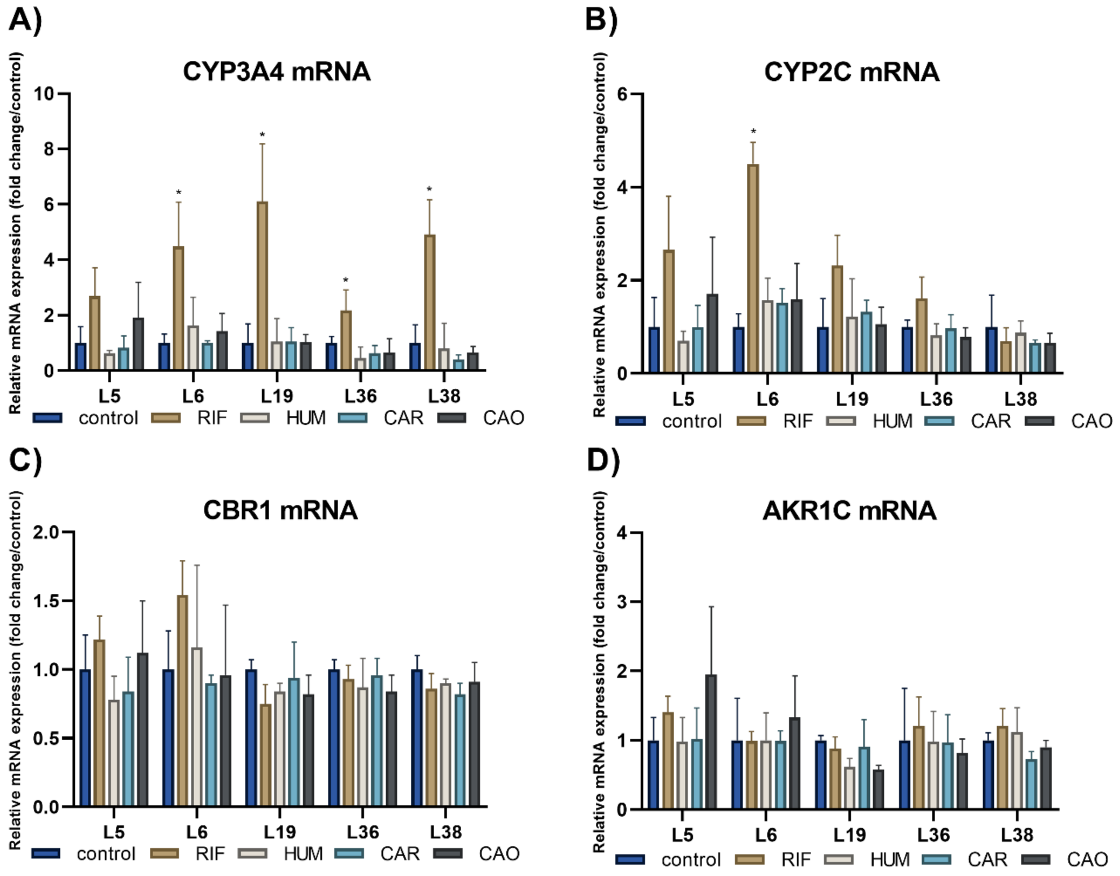

Figure S1. Inter-individual differences in the effect of cyclic sesquiterpenes (10 μM) and RIF (10 μM) on the normalized mRNA expression of CYP3A4 (**A**), CYP2C (**B**), CBR1 (**C**) and AKR1C (**D**) in human PCLS from five patients after 24 h (n = 3). The normalized expression level was calculated using the $2^{-\Delta\Delta Ct}$ method with the geometric mean of GAPDH and SDHA as a reference gene. Results are presented as the mean ± SD (n = 3). Statistical analyses were performed using one-way ANOVA with Dunnett's test: $p < 0.05$ (*).

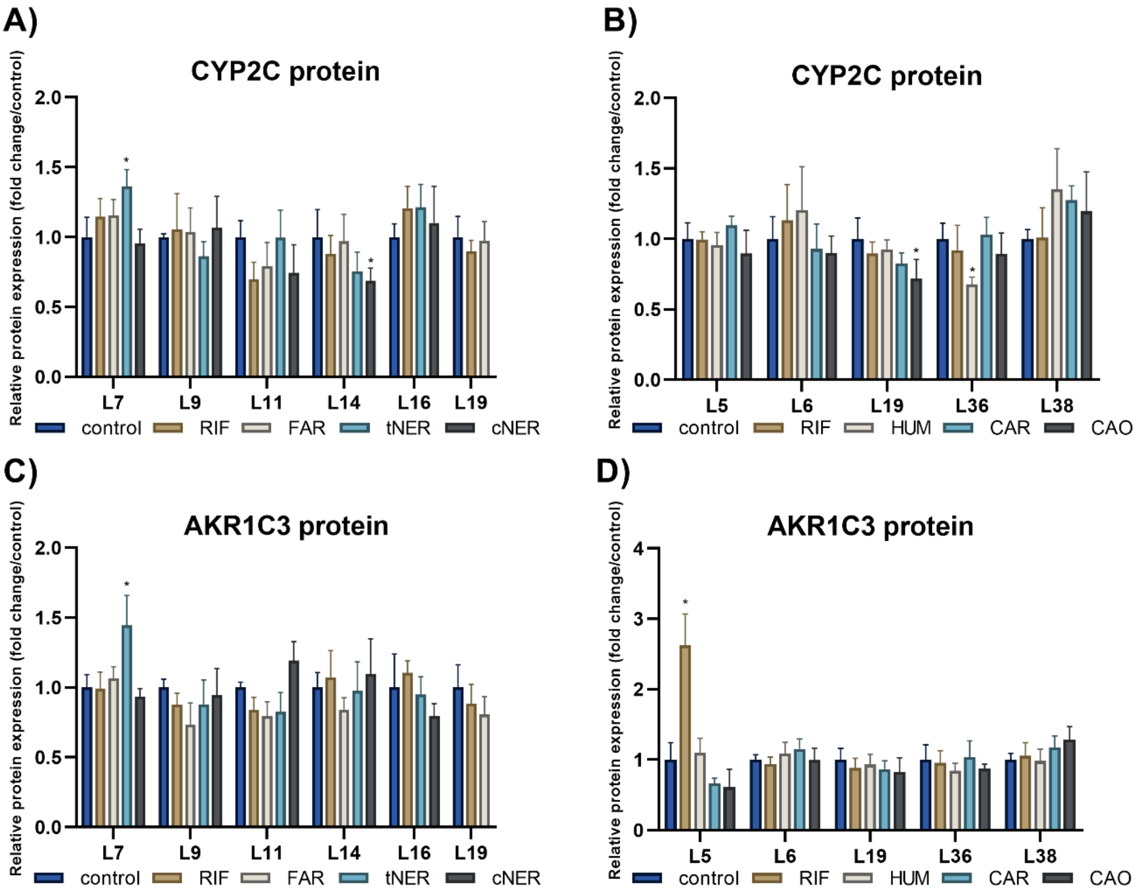

Figure S2. Inter-individual differences in the effect of sesquiterpenes (10 μM) and RIF (10 μM) on the normalized protein expression of CYP2C (**A**, **B**) and AKR1C3 (**C**, **D**) in human PCLS from ten patients after 24 h (n = 3). The protein expression was calculated using calnexin as a loading control. Results are presented as the mean ± SD (n = 4), with controls set to 100%. Statistical analyses were performed using one-way ANOVA with Dunnett's test: $p < 0.05$ (*).
